# Tigecycline Versus Colistin in the Treatment of Carbapenem-resistant *Acinetobacter baumannii* Complex Osteomyelitis

**DOI:** 10.7150/jbji.42448

**Published:** 2020-02-21

**Authors:** Priscila R. Oliveira, Vladimir C. Carvalho, Eduardo S. Saconi, Marcos C. Leonhardt, Kodi E. Kojima, Jorge S. Santos, Flavia Rossi, Ana Lucia L.M. Lima

**Affiliations:** 1 Instituto de Ortopedia e Traumatologia, Hospital das Clinicas HCFMUSP, Faculdade de Medicina, Universidade de São Paulo, SP, Brazil;; 2 Laboratorio de Microbiologia DLC, Hospital das Clinicas HCFMUSP, Faculdade de Medicina, Universidade de São Paulo, SP, Brazil

**Keywords:** *Acinetobacter*, carbapenem-resistant, colistin, osteomyelitis, tigecycline

## Abstract

**Background**: *Acinetobacter baumannii* complex is an increasingly important cause of osteomyelitis. It is considered a difficult to treat agent, due to increasing antimicrobial resistance and few available therapeutic options.

**Objective**: To compare effectiveness and tolerability of tigecycline and colistin in patients with osteomyelitis caused by carbapenem-resistant *A. baumannii* complex (CRABC).

**Methods**: This retrospective review included all patients admitted to a 150-bed tertiary hospital from 2007 to 2015 with microbiologically confirmed CRABC osteomyelitis for which they received tigecycline or colistin. Data on demographic and clinical characteristics, adverse events, and outcomes 12 months after the end of antimicrobial treatment were analysed and stratified according to the antimicrobial used.

**Results**: 65 patients were included, 34 treated with colistin and 31 with tigecycline. There were significantly more men (*P* = 0.028) in the colistin group, and more smokers (*P* = 0.021) and greater occurrence of chronic osteomyelitis (*P* = 0.036) in the tigecycline treatment group. Median duration of therapy was 42.5 days for colistin and 42 days for tigecycline, with no significant difference. Overall incidence of adverse events was higher in the colistin group (*P* = 0.047). In particular, incidence of renal impairment was also higher in this group (*P* = 0.003). Nausea and vomiting were more frequent with tigecycline (*P* = 0.046). There were no significant differences between groups in relapse, amputation, or death.

**Conclusions**: Tigecycline had a better safety profile than colistin in the treatment of osteomyelitis due to CRABC, with no significant difference in outcomes after 12 months of follow-up.

## 1. Introduction

Infections related to *Acinetobacter baumannii* complex are a challenge in current clinical practice, particularly because they are often antibiotic resistant. The emergence of multidrug-resistant (MDR) and extensively drug-resistant (XDR) strains not susceptible to carbapenems further complicates treatment.^1^ In 2017, the World Health Organization identified strains of *A. baumannii*, *Pseudomonas aeruginosa* and carbapenem-resistant Enterobacteriales as priority pathogens for the development of new and improved treatment strategies.^2^

In addition to being an important cause of healthcare-associated infections such as pneumonia and urinary tract infections, *A. baumannii* complex has become an increasingly important cause of osteomyelitis, particularly in cases with a traumatic aetiology.^3^,^4^ Case reports of post-traumatic osteomyelitis treated in civilian and combat-related environments on different continents implicate *A. baumannii* complex as a frequent cause of bone infections.^5^-^7^ Some reports note the prevalence of highly antimicrobial resistant strains.^8^-^11^ There are few effective therapeutic options for carbapenem-resistant *A. baumannii* complex osteomyelitis, primarily due to less than optimal antimicrobial penetration into bone tissue.^12^

Tigecycline has good penetration into bone tissue and is active against some carbapenem-resistant *A. baumannii* complex strains.^13^ As such, it appears to be a promising option for patients with carbapenem-resistant *A. baumannii* complex osteomyelitis. Some case reports show therapeutic success, but there are no studies comparing the use of tigecycline with colistin, another therapeutic option used in such situations.^14^,^15^

The objective of this retrospective analysis was to compare the effectiveness, safety, and outcomes of tigecycline versus colistin in patients with osteomyelitis caused by carbapenem-resistant *A. baumannii* complex in a setting with an unusually high number of osteomyelitis cases caused by carbapenem MDR and XDR *A. baumannii* complex.

## 2. Material and Methods

This observational, retrospective cohort study was conducted at the *Instituto de Ortopedia e Traumatologia do Hospital das Clínicas da Faculdade de Medicina da Universidade de São Paulo*, a 150-bed hospital that specialises in complex orthopaedic and traumatology cases. The study was approved by the Institution's Ethics Committee and is registered on clinicatrials.gov (NCT03559530).

### 2.1. Case Selection and Data Analysis

Cases were selected from the institutional Infection Control Board database among all patients with microbiologically confirmed *A. baumannii* complex osteomyelitis confirmed by positive bone tissue culture results. Antimicrobial susceptibility test profiles were used to verify whether the strain isolated from each patient met the criteria for MDR or XDR classification.^1^

The study included patients who received tigecycline 50 mg intravenous every 12 hours or colistin (colistin base activity) 2.5 mg/kg intravenous every 12 hours for the treatment of carbapenem-resistant *A. baumannii* complex osteomyelitis from January 2007 to December 2015. All patients were required to have received loading doses of each agent and had confirmed infection, as demonstrated by extensive microbiologic analysis of cultured bone tissue. To avoid bias, all patients who completed at least 24 hours of the chosen antimicrobial were included and kept in the study for analysis. No other antimicrobial agents have been used in combination to treat *A. baumannii* complex infection.

The choice of therapy for patients included in this study was at the discretion of the attending physician. Cases in which tigecycline and colistin were used simultaneously were excluded. If colistin and tigecycline were used sequentially, the patient was included in the treatment group according to the drug that was used longer. For patients receiving colistin and worsening renal function, the dose of this antimicrobial was corrected for creatinine clearance if the attending physician decided to maintain this antimicrobial.

The following demographic and clinical characteristics of patients in each treatment group were assessed: sex; age; length of hospital stay; basal creatinine level; Charlson´s index; physical status classification according to American Society of Anaesthesiologists' score (ASA score); presence of comorbities or immunosuppression; history of illicit drug use, active smoking or alcohol abuse; previous surgery on affected limb; osteomyelitis classification according to possible origin and duration; infections related to pressure ulcer; implant-related infection (infection directly related to the presence of the implant, in the same topography) and need to implant removal during treatment (removal for any cause, not just infection related); number of surgical procedures for treatment; need for soft tissue repair; use of negative pressure therapy; previous antimicrobial use and duration of treatment with colistin or tigecycline.

Adverse events reported in the medical record within 48 hours after initiation of therapy with colistin or tigecycline until the end of each treatment were analysed, including: renal impairment (defined as a 1.5-fold increase in serum creatinine in relation to the start of treatment);^16^ liver enzyme alterations; nausea and vomiting; and skin rash. Any other adverse event that occurred during treatment, and was considered by the attending physician to be related to the antimicrobial that was used, was classified as “other event”.

Due to the high recurrence rate of osteomyelitis, clinical outcomes were evaluated 12 months after the end of antimicrobial treatment. Patients who did not present with signs of recurrent infection were considered to have achieved remission rather than cure.^17^ The following classifications were used to evaluate outcomes: remission of signs and symptoms of infection, recurrence of infection, amputation of the affected limb, and death. To categorise the outcomes, “favourable” was defined as remission of infection, while “unfavourable” was defined as relapse, amputation, or death.

### 2.2. Microbiological Analysis

Consistent with the institutional protocol that was in effect throughout the study period, bone tissue cultures were collected at the time of surgery following extensive debridement of the devitalised tissues. Samples were placed in sterile vials containing thioglycolate medium and sent for laboratory analysis. Positive samples were identified through automated VITEK2 and matrix-assisted laser desorption/ionization time-of-flight mass spectrometry systems. Antibiotic susceptibility testing was done using the VITEK2 system with a card customised for Brazil that included colistin. The breakpoints used in categorical interpretation for all antimicrobials (with the exception of tigecycline) were those effective each year according to the Clinical and Laboratory Standards Institute M100 document; quality control was performed according to the manufacturer's instructions. To assess the sensitivity of *A. baumannii* complex isolates to tigecycline, breakpoints were inferred from available recommended values for Enterobacteriales.^18^, ^19^

### 2.3. Statistical Analyses

Quantitative variables were described using summary measures (mean and standard deviation or median) and compared between groups using Student's t-tests or Mann-Whitney tests. Qualitative variables were described using absolute and relative frequencies. The existence of association between variables was verified with chi-square tests or exact tests (Fisher's exact test or likelihood ratio test). Kaplan-Meier function was estimated according to groups to assess the occurrence of recurrence in patients and the incidence of recurrence was compared between groups using the log-rank test. For this particular analysis, deaths were excluded, as practically all deaths occurred during hospitalization, that is, without risk of recurrence. The time intervals for recurrence were described using the mean and standard deviation. IBM-SPSS for Windows version 20.0 software was used to do the analyses. Data were tabulated using Microsoft Excel 2003 software. The tests were performed with a significance level of 5%.

## 3. Results

**Figure 1:**
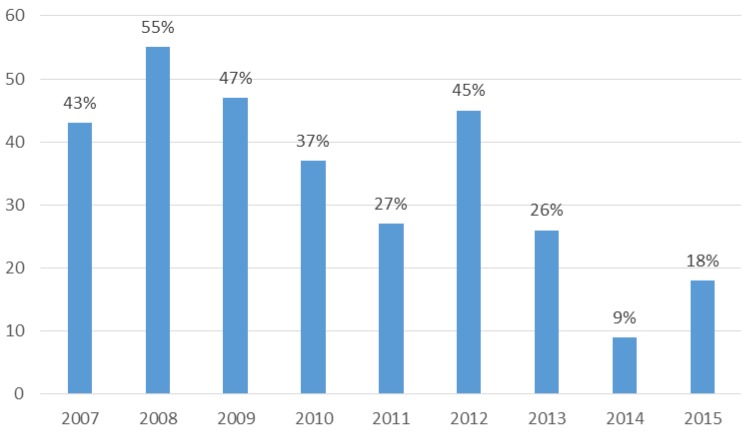
Temporal evolution of the carbapenem susceptibility profile of *A. baumannii* complex strains causing osteomyelitis during the study period.

A total of 171 patients with *A. baumannii* complex osteomyelitis were treated at our hospital during the study period. The susceptibility of these isolates to carbapenems declined substantially over time, ranging from 55% in 2008 to 18% in 2015 (Figure 1). Some between-year variability was observed, with only 9% of isolates susceptible to carbapenems in 2014.

**Figure 2:**
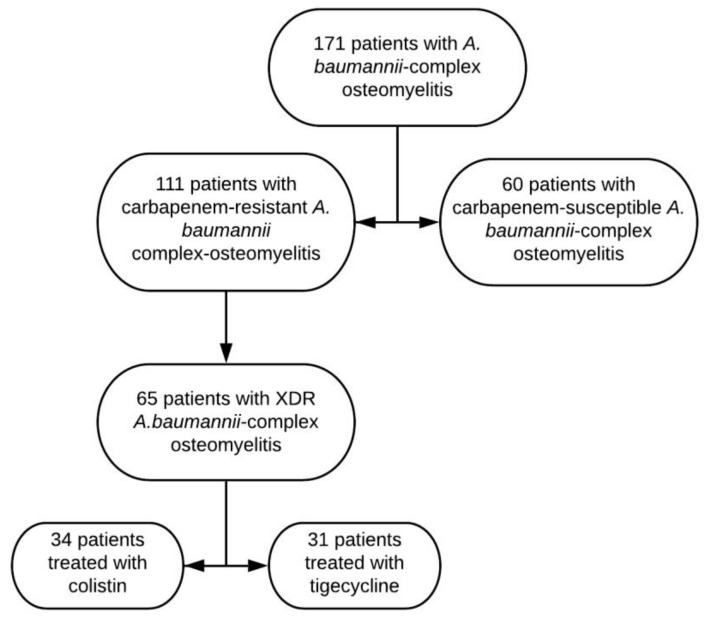
Chart flow with the distribution of all patients treated with *A. baumannii* complex osteomyelitis and those selected for the present study

In this setting of high multidrug resistance, 65 patients with osteomyelitis had infection caused by *A. baumannii* complex strains defined as XDR, as demonstrated by the minimum inhibitory concentrations (MICs) for the antimicrobials tested (Appendix). Of these 65 patients, 34 were treated with colistin and 31 with tigecycline, no other antibiotics were used to cover *A. baumannii* in combination (Table 1). The chart flow with the distribution of all patients treated with *A. baumannii* complex osteomyelitis and those selected for the present study is shown in figure 2. Presence of co-infection with other agents (polymicrobiota) was present in 63 of the 65 patients included in the study (96.9%). Patients in the two treatment groups were generally well matched, although there were significantly more men in the colistin treatment group (85.3%) than in the tigecycline group (61.3%) (*P* = 0.028). On the other hand, there were significantly more smokers in the tigecycline group (16.1% versus zero in colistin group, *P* = 0.021), as well as more patients with chronic osteomyelitis (61.3% versus 35.3% in colistin group, *P* = 0.036). Most cases were post-traumatic osteomyelitis in both groups, and there was no difference in the number of patients with of open fracture in each group. All open fractures in both groups were classified as Gustilo 3. There were no significant differences between groups regarding the other variables analysed, including the duration of antimicrobial therapy or the number of surgical procedures performed for the treatment of osteomyelitis. Only one patient in the tigecycline-treated group had previously received colistin. No colistin-treated patients received tigecycline prior to the use of this drug.

The incidence of renal impairment was significantly higher among patients treated with colistin (58.8% versus 22.6% in tigecycline group, *P* = 0.003). The overall incidence of adverse events was also higher for colistin-treated patients (67.6% versus 41.9% in tigecycline group, *P* = 0.047). The incidence of nausea and vomiting was higher in the tigecycline treatment group (12.9% versus zero in colistin group, *P* = 0.046) (Table 2). For three patients, the occurrence of adverse events led to switch of therapy: two patients receiving colistin who presented renal impairment and one patient receiving tigecycline who presented unavoidable vomiting.

**Figure 3:**
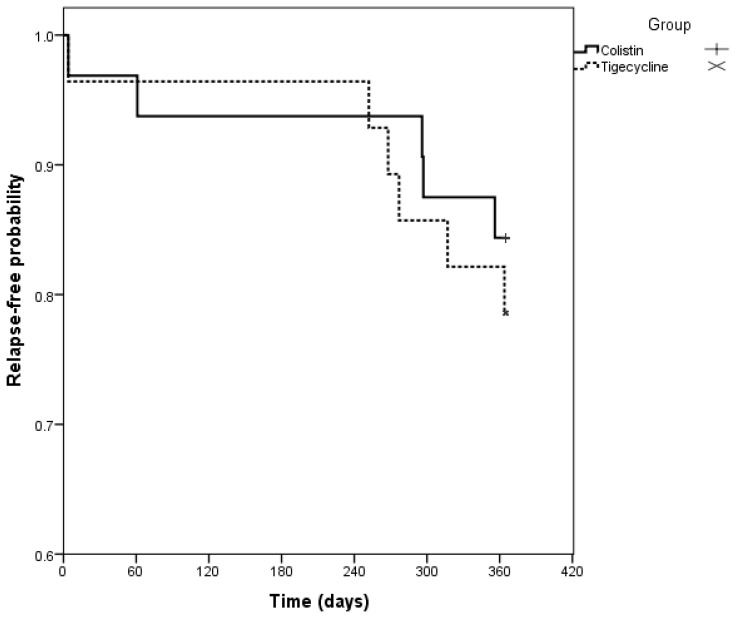
Kaplan-Meier function of recurrence time according to treatment groups.

The outcomes observed 12 months after cessation of antimicrobial therapy are shown in Tables 3 and 4. At the end of the follow-up period, 44.1% of colistin-treated patients and 38.7% of tigecycline-treated patients had no signs or symptoms of infection, and were considered to be in remission. There was a higher incidence of patients requiring amputation in the colistin-treated group (20.6% versus 6.45% in tigecycline group) and a greater loss of follow-up among tigecycline-treated patients (25.8% versus 1.7% in colistin group). However, no statistically significant differences in these outcomes were noted (*P* = 0.433). As shown in figure 3, the Kaplan Meier function estimate did not suggest any difference in the time intervals of recurrence between the groups, confirmed by the use of the log-rank test (*P* = 0.569). Among the patients who relapsed, the average time of relapse was 227 days with a standard deviation of 136 days.

When the outcomes were categorically analyzed as favorable (remission), unfavorable (recurrence, amputation or death) or lost to follow up, there was no significant difference between groups either (*P* = 0.535). Among the cases with unfavorable outcome, the development of antimicrobial resistance was not reported.

## 4. Discussion

The increasing incidence of *A. baumannii* complex osteomyelitis caused by carbapenem-resistant strains represents a challenge in current clinical practice.^20^ Unlike other Gram-negative bacilli that are frequently resistant to carbapenems, such as *P. aeruginosa* or *Klebsiella pneumoniae*, there are no new therapeutic options that effectively target *A. baumannii* complex. Tigecycline and polymyxins, an antibiotic group that includes colistin and polymyxin B, remain the main treatment options for infections caused by MDR *A. baumannii* complex.^21^

Adverse events that occur during osteomyelitis treatment are quite limiting due to the length of the recommended treatment periods, which can reach 6 months.^22^ The use of colistin is especially limiting in this scenario, due to the high potential for nephrotoxicity.^23^ In addition, the ability of colistin to penetrate bone tissue has not been established. Tigecycline has good bone penetration and is associated with fewer clinically serious adverse events such as renal events, and these renal events appear to drive the higher overall adverse events with colistin. Tigecycline is therefore a reasonable option for treatment of bone infections associated with carbapenem-resistant Gram-negative bacilli.^24^ Experience with tigecycline in this setting is somewhat limited, as it is not formally indicated for MDR *A. baumannii* complex osteomyelitis.

In the present study, the use of tigecycline to treat patients with osteomyelitis due to carbapenem-resistant *A. baumannii* complex was associated with a lower overall occurrence of adverse events than treatment with colistin. Tigecycline was also associated with significantly less renal impairment than colistin. Although nausea and vomiting were more frequent in patients receiving tigecycline, this adverse event was not associated with severe outcomes in any patient included in the present study and is known to be related to the use of tigecycline.^25^

There were no significant differences in outcomes between the two groups. However, it should be noted that the proportion of smokers and patients with chronic osteomyelitis was higher in patients treated with tigecycline, and smoking is generally associated with a worse prognosis in the treatment of bone and joint infections.^26^ The observed remission rate of 41.5% was lower than that described for patients who are at least 6 months post-treatment for osteomyelitis in general (69% to 72%), and for patients with Gram- negative pathogens (60%).^27^,^28^ These data corroborate the difficulty of treating osteomyelitis related to carbapenem-resistant *A. baumannii* complex. This scenario may be even worse for implant-related infections. 29 In the studied sample, there was no statistically significant difference between the number of patients with implants and the need for removal of these devices for treatment, but it must be considered that the total number of patients with infection directly related to orthopedic implant was small (20% of total patients).

One of the limitations of this study is related to the follow-up period of the patients, which was 12 months and may be considered short for the evaluation of osteomyelitis. Treated cases of these infections may recur years after treatment has ended. In addition, particular mechanisms of *A. baumannii* complex such as biofilm production and glycoconjugate formation could also be related to cases of late relapse of infection. 17,30

Another important limitation of this study is that the automated colistin susceptibility testing system may produce false susceptible results, as has been described in the literature. Colistin antibiotic susceptibility test results found by automated systems to be within the susceptible range, particularly those at the susceptibility breakpoint (2 mg/L), should be validated by broth microdilution, but this method is not readily available.^31^ However, the great majority of the isolates described in this study had MIC values <1 mg/L, which favours the accuracy of the susceptibility results found. Other clinical studies using colistin for the treatment of osteomyelitis are necessary to validate this finding. Future studies are also needed to assess the safety and efficacy of the combined use of colistin and tigecycline in this setting of *A. baumannii* complex related osteomyelitis with a high profile of antimicrobial resistance and which present high rates of infection recurrence after treatment.

## 5. Conclusion

Data analysis from this retrospective study showed that tigecycline was associated with a better safety profile than colistin for the treatment of osteomyelitis caused by carbapenem-resistant *A. baumannii* complex, with no significant difference in outcomes after 12 months of follow-up.

## Supplementary Material

Appendix.

## Figures and Tables

**Table 1:** Distribution of patients with carbapenem-resistant A. baumannii complex osteomyelitis according to demographic and clinical characteristics and comparison according to treatment group (colistin or tigecycline)

Demographic and clinical characteristics	Treatment group		Total (N =65)	*P*
	Colistin (N = 34)	Tigecycline (N = 31)		
Sex (male)	29 (85.3)	19 (61.3)	48 (73.8)	0.028
Age (mean years ± SD)	40.6 ± 19.1	46.8 ± 18.9	43.6 ± 19.1	0.193^c^
Median length of hospital stay (range of days)	74.5 (13; 331)	64 (0; 226)	70 (0; 331)	0.948^d^
Mean basal creatinine level (± SD)	0.69 ± 0.36	0.82 ± 0.54	0.75 ± 0.45	0.260^c^
Mean Charlson's index (± SD)	0 (0; 5)	1 (0; 7)	0 (0; 7)	0.083^d^
ASA score (%)				0.189
I	11 (32.4)	8 (25.8)	19 (29.2)	
II	20 (58.8)	15 (48.4)	35 (53.8)	
III	3 (8.8)	8 (25.8)	11 (16.9)	
Presence of comorbidities (%)	10 (29.4)	13 (41.9)	23 (35.4)	0.292
Systemic hypertension	6 (17.6)	9 (29)	15 (23.1)	0.277
Neoplasia	2 (5.9)	3 (9.7)	5 (7.7)	0.663^a^
Diabetes	4 (11.8)	2 (6.5)	6 (9.2)	0.674^a^
HIV infection	0 (0)	2 (6.5)	2 (3.1)	0.224^a^
Rheumatoid arthritis	2 (5.9)	1 (3.2)	3 (4.6)	>0.999^a^
Other rheumatic diseases	0 (0)	1 (3.2)	1 (1.5)	0.477^a^
Immunosuppression (%)	1 (2.9)	1 (3.2)	2 (3.1)	>0.999^a^
Illicit drug use (%)	0 (0)	1 (3.2)	1 (1.5)	0.477^a^
Active smoking (%)	0 (0)	5 (16.1)	5 (7.7)	0.021^a^
Alcohol abuse (%)	2 (5.9)	4 (12.9)	6 (9.2)	0.413^a^
Previous surgery on affected limb (%)	10 (29.4)	14 (45.2)	24 (36.9)	0.189
Affected limb (%)				0.414
Lower limbs	29 (85.3)	24 (77.4)	53 (81.5)	
Upper limbs	5 (14.7)	7 (22.6)	12 (18.5)	
Classification of osteomyelitis according to possible origin (%)				0.841^b^
Post-traumatic	23 (67.6)	19 (61.3)	42 (64.6)	
Contiguity	7 (20.6)	9 (29)	16 (24.6)	
Haematogenic	2 (5.9)	2 (6.5)	4 (6.2)	
Spine	2 (5.9)	1 (3.2)	3 (4.6)	
Open fracture (%)	17 (50)	10 (32,3)	27 (41,5)	0,147
Classification of osteomyelitis according to duration (%)				0.036
Acute	22 (64.7)	12 (38.7)	34 (52.3)	
Chronic	12 (35.3)	19 (61.3)	31 (47.7)	
Infection related to pressure ulcer (%)	6 (17.6)	4 (12.9)	10 (15.4)	0.736^a^
Presence of implant (%)	6 (17.6)	3 (9.7)	9 (13.8)	0.480^a^
Need to remove implant (%)	4 (11.8)	9 (29)	13 (20)	0.082
Median number of surgical procedures for treatment (range)	3 (1; 12)	3 (1; 9)	3 (1; 12)	0.510^d^
Need for soft tissue repair (%)	10 (29.4)	8 (25.8)	18 (27.7)	0.746
Use of negative pressure therapy (%)	12 (35.3)	6 (19.4)	18 (27.7)	0.151
Previous antimicrobial use (%)	34 (100)	30 (96.8)	64 (98.5)	0.477^a^
Median duration of treatment (range of days)	42.5 (1; 193)	42 (9; 193)	42 (1; 193)	0.438^d^
				

SD=standard deviation The Chi-square test was used for statistical analyses, unless otherwise indicated. **^a^**Fisher's exact test; **^b^**Likelihood ratio test; **^c^**Student's t-test; **^d^**Mann-Whitney test.

**Table 2:** Comparison of incidence of adverse events during treatment for patients receiving colistin or tigecycline

Adverse events	Treatment group		Total (N = 65)	*P* value
	Colistin (N = 34)	Tigecycline (N = 31)		
Overall adverse events	23 (67.6)	13 (41.9)	36 (55.3)	0.047^a^
Renal impairment	20 (58.8)	7 (22.6)	27 (41.5)	0.003^ a^
Liver enzymes abnormalities	1 (2.9)	1 (3.2)	2 (3.1)	>0.999^b^
Nausea and vomiting	0 (0)	4 (12.9)	4 (6.2)	0.046^b^
Skin rash	1 (2.9)	0 (0)	1 (1.5)	>0.999^b^
Others	5 (14.7)	2 (6.4)	7 (10.7)	0.430^ a^

Data expressed as n (%) **^a^**Chi-square test **^b^**Fisher's exact test

**Table 3:** Distribution of the outcomes observed after 12 months of treatment according to the antimicrobial used

Outcome	Treatment group		Total (N = 65)	*P* value
	Colistin (N = 34)	Tigecycline (N = 31)		
Remission^a^	15 (44.1)	12 (38.7)	27 (41.5)	0.433^b^
Death	2 (5.8)	3 (9.6)	7 (7.7)	
Recurrence of *A. baumannii* osteomyelitis after treatment	5 (14.7)	6 (19.3)	11 (16.9)	
Amputation	7 (20.6)	2 (6.45)	9 (13.8)	
Lost to follow up	5 (1.7)	8 (25.8)	13 (20)	

Data expressed as n (%) **^a^**Patients who did not present with signs of recurrent infection were considered to have achieved remission. **^b^**Fisher's exact test was used

**Table 4:** Categorised distribution of outcomes after 12 months of treatment according to the antimicrobial used.

Outcome	Treatment group		Total (N = 65)	*P* value
	Colistin (N = 34)	Tigecycline (N = 31)		
Unfavourable^a^	14 (41.2)	11 (35.5)	25 (38.5)	0.535^b^
Favourable	15 (44.1)	12 (38.7)	27 (41.5)	
Lost to follow up	5 (14.7)	8 (25.8)	13 (20)	

Data expressed as n (%) **^a^**Defined as relapse, amputation or death **^b^**Chi-squared test
