# Supplementary material for: Tigecycline Versus Colistin in the Treatment of Carbapenem-resistant Acinetobacter baumannii Complex Osteomyelitis
Source: J Bone Jt Infect. 2020 Feb 21;5(2):60–6. doi: 10.7150/jbji.42448 (PMC7242406; doi:10.7150/jbji.42448)
Supplement: Supplementary file 1 — Appendix. [file jbjiv05p0060s1.pdf]

APPENDIX. MIC values for the main antimicrobials tested for isolates of *A. baumannii* complex causing osteomyelitis for patients included in the study, illustrating that all isolates are XDR. Order of the line classification follows the order of patients' inclusion in the study.

**Treatment Group**	**MIC values (mg/L)**									
	**Amikacin**	**Ampicillin Sulbactam**	**Cefepime**	**Ceftazidime**	**Ciprofloxacin**	**Imipenem**	**Meropenem**	**Gentamycin**	**Tigecycline**	**Colistin**
**Colistin**	16	>32	>64	>64	>4	>16	>16	2	2	<0.5
**Tigecycline**	16	16	>64	>64	>4	>16	>16	4	2	<0.5
**Colistin**	32	>32	>64	>64	>4	>16	>16	<1	1	<0.5
**Colistin**	<2	16	>64	>64	>4	>16	>16	2	2	<0.5
**Colistin**	16	16	>64	>64	>4	>16	>16	2	4	<0.5
**Colistin**	16	>32	>64	>64	>4	>16	>16	>16	4	<0.5
**Tigecycline**	32	16	>64	>64	>4	>16	>16	4	2	<0.5
**Colistin**	16	>32	>64	>64	>4	>16	>16	>16	1	<0.5
**Colistin**	16	>32	>64	>64	>4	>16	>16	>16	4	<0.5
**Colistin**	8	16	>64	>64	>4	>16	>16	<1	8	<0.5

**Tigecycline**	>64	>32	>64	>64	<4	>16	>16	4	4	<0.5
**Colistin**	32	>32	>64	>64	>4	>16	>16	4	2	<0.5
**Colistin**	32	>32	>64	>64	>4	>16	>16	4	2	<0.5
**Colistin**	32	>32	>64	>64	>4	>16	>16	>16	8	1
**Colistin**	16	>32	>64	>64	>4	>16	>16	>16	0,5	<0.5
**Tigecycline**	16	16	>64	>64	>4	>16	>16	>16	4	<0.5
**Tigecycline**	16	>32	>64	>64	>4	>16	>16	>16	0,5	<0.5
**Colistin**	16	>32	>64	>64	>4	>16	>16	4	2	<0.5
**Colistin**	16	16	>64	>64	>4	>16	>16	>16	2	<0.5
**Tigecycline**	16	16	>64	>64	>4	>16	>16	4	2	<0.5
**Tigecycline**	16	16	>64	>64	>4	>16	>16	1	1	<0.5
**Colistin**	16	>32	>64	>64	>4	>16	>16	2	4	<0.5
**Tigecycline**	32	>32	>64	>64	>4	>16	>16	4	2	<0,25
**Colistin**	16	16	>64	>64	>4	>16	>16	1	1	<0.5
**Tigecycline**	16	16	>64	>64	>4	>16	>16	8	2	<0.5
**Colistin**	16	>32	>64	>64	>4	>16	>16	2	2	<0.5

**Colistin**	>64	>32	>64	>64	>4	>16	>16	>16	4	<0.5
**Tigecycline**	16	16	>64	>64	>4	>16	>16	2	2	0,25
**Tigecycline**	16	>32	>64	>64	>4	>16	>16	>16	4	1
**Colistin**	<2	>32	>64	>64	>4	>16	>16	4	>8	<0.5
**Tigecycline**	16	16	>64	>64	>4	>16	>16	2	2	<0,25
**Colistin**	>64	>32	>64	>64	>4	>16	>16	>16	4	<0.5
**Tigecycline**	16	16	>64	>64	>4	>16	>16	4	2	<0.5
**Colistin**	16	16	>64	>64	>4	>16	>16	<1	<0.5	<0.5
**Tigecycline**	16	16	>64	>64	>4	>16	>16	>16	4	<0.5
**Tigecycline**	16	16	>64	>64	>4	>16	>16	>16	2	<0.5
**Tigecycline**	>64	8	16	>64	>4	>16	>16	>16	2	<0.5
**Tigecycline**	>64	16	>64	8	>4	>16	>16	>16	<0.5	>16
**Tigecycline**	<2	16	>64	>64	>4	16	>16	>16	<0.5	<0.5
**Colistin**	>64	16	>64	>64	>4	>16	>16	2	4	<0.5
**Tigecycline**	>64	>32	>64	>64	>4	>16	>16	4	2	<0.5
**Tigecycline**	16	16	>64	>64	>4	>16	>16	2	2	<0.5

**Tigecycline**	8	>32	>64	>64	>4	>16	>16	>16	<0.5	<0.5
**Colistin**	16	8	>64	>64	>4	>16	>16	2	2	<0.5
**Colistin**	16	16	>64	>64	>4	>16	>16	4	2	<0.5
**Tigecycline**	>64	>32	>64	>64	>4	>16	>16	8	2	<0.5
**Tigecycline**	16	>32	>64	>64	>4	>16	>16	>16	4	1
**Colistin**	16	8	>64	>64	>4	<16	>16	4	NT*	<0.5
**Colistin**	16	>32	>64	>64	>4	>16	>16	>16	1	<0.5
**Tigecycline**	16	>32	>64	>64	>4	>16	>16	>16	1	<0.5
**Colistin**	>64	>32	>64	>64	>4	>16	>16	>16	>8	<0.5
**Colistin**	>64	>32	>64	>64	>4	>16	>16	4	4	<0.5
**Colistin**	>64	>32	>64	>64	>4	>16	>16	>16	1	<0.5
**Tigecycline**	16	16	>64	>64	>4	>16	>16	>16	2	<0.5
**Colistin**	16	16	>64	>64	>4	>16	>16	>16	2	<0.5
**Tigecycline**	16	16	>64	>64	>4	>16	>16	>16	2	<0.5
**Tigecycline**	16	16	>16	>16	>4	>16	>16	8	2	<0.5
**Tigecycline**	32	32	>64	>64	>4	>16	>16	>16	2	1

**Colistin**	<2	>32	>64	>64	>4	>16	>16	>16	2	<0.5
**Tigecycline**	16	>32	>64	>64	>4	>16	>16	>16	<0.5	<0.5
**Colistin**	16	16	>64	>64	>4	>16	>16	>16	<0.5	<0.5
**Colistin**	16	>32	>64	>64	>4	>16	>16	>16	4	1
**Colistin**	<2	>32	>64	4	>4	>16	>16	<1	<0.5	<0.5
**Colistin**	16	>32	>64	>64	>4	>16	>16	>16	<0.5	<0.5
**Tigecycline**	16	>32	>64	>64	>4	>16	>16	<1	1	<0.5
**Colistin**	<2	>32	>64	>64	>4	>16	>16	<1	1	<0.5
